# Proportional Integral Derivative Control in Spark Plasma Sintering Simulations

**DOI:** 10.3390/ma14071779

**Published:** 2021-04-03

**Authors:** Runjian Jiang, Elisa Torresani, Guodong Cui, Eugene A. Olevsky

**Affiliations:** 1 Powder Technology Laboratory, San Diego State University, San Diego, CA 92182, USA; etorresani@sdsu.edu (E.T.); eolevsky@sdsu.edu (E.A.O.); 2 School of Materials Science and Engineering, Southwest Jiaotong University, Chengdu 610031, China; gdcui@swjtu.edu.cn

**Keywords:** proportional integral derivative, spark plasma sintering, temperature regulation, heating responsiveness

## Abstract

The prediction of microstructure evolution and densification behavior during the spark plasma sintering (SPS) process largely depends on accurate temperature regulation. A loop feedback control algorithm called proportional integral derivative (PID) control is a practical simulation tool, but its coefficients are often determined by an inefficient “trial and error” method. This paper is devoted to proposing a numerical method based on the principles of variable coefficients to construct an optimal linear PID controller in SPS electro-thermal simulations. Different types of temperature profiles were applied to evaluate the feasibility of the proposed method. Simulation results showed that, for temperature profiles conventionally used in SPS cycles, the PID output keeps pace with the desired profile. Characterized by an imperfect time delay and overshoot/undershoot, the constructed PID controller needs further advancement to provide a more satisfactory temperature regulation for non-continuous temperature profiles. The first step towards a numerical rule for the optimal PID controller design was undertaken in this work. It is expected to provide a valuable reference for the advanced electro-thermal modeling of SPS.

## 1. Introduction

As an advanced field-assisted sintering technique, spark plasma sintering (SPS) has been widely applied to rapidly densify a variety of materials [1,2,3]. The pulsed electric current allows this process to attain a higher temperature (above 2300 K), higher heating rate (up to 200 K/min) and shorter duration (only a few minutes) than other conventional sintering tools [4]. These outcomes permit the significant grain growth inhibition and the retention of fine microstructure from raw powders [5]. The high densification of refractory materials [6], composite materials [7] and other novel functional materials [8] can be achieved.

The SPS process should be controllable and reproducible; however, a non-uniform temperature spatial distribution is often generated within the die and the sample [9,10,11]. One major challenge of the SPS technique is to understand the electro-thermal coupled phenomena in the die–sample setup and their evolution over time [12]. Stable temperature regulation is of particular importance. The loop feedback control algorithm called proportional integral derivative (PID) control is often used for adjusting the temperature variation during SPS [13]. In this way, the finite element method (FEM) can be a powerful tool to better establish the electro-thermal features during the SPS process [14,15]. Paired with the linear PID controller, the FEM is a perfect simulation instrument for reproducing the temperature cycle and predicting the multi-physical features of the SPS process.

The classic linear PID controller often contains three unknown coefficients (or gains) that should be determined. So far, there has not been a unified specification for them in SPS simulations, while PID regulations contrast for different tooling dimensions and physical conditions. Normally, simulators follow the “trial and error” guideline in FEM practice, which involves many time-consuming and inefficient procedures. The “trial and error” method, to some extent, means no predefined rules. It is an undesired step reducing the regulation accuracy in the FEM simulations. Placing the PID control in the maximum responsiveness area, which is the mid-height of the punches, is a typical approach improving the temperature regulation efficiency [16]. In spite of that, the non-optimal initial PID coefficients prevent obtaining a satisfactory level of the process control. Nowadays, advanced SPS technologies are moving towards more diverse application scenarios, requiring precise controls of the densification behavior and microstructure evolution; hence, more complex SPS temperature profiles are frequently utilized. In these cases, the searching for the optimal PID coefficients based on a “trial and error” method becomes unreliable. Isn’t there a feasible criterion for PID-based temperature regulation in SPS?

The design of a PID controller is often affected by the input signal in the controlled object. For the SPS process, the temperature of the PID control area is requested to follow the prescribed cycle; thus, the type of the prescribed temperature profiles in the sintering setup should be treated carefully. Their characteristics, for instance the temperature and heating rate over time, may determine the coefficients used to construct different PID controllers. Considering these factors, this paper describes the development of an appropriate rule defining the optimal linear PID controller to improve the temperature regulation in the SPS simulations. Two kinds of temperature profiles were assessed in the present study: continuous profiles where temperature changes gradually over time and non-smooth profiles where temperature changes suddenly. A more precise temperature regulation process was provided with the proposed framework based on the principle of variable PID coefficients. In this study, the first step towards a rule for producing the optimal PID controller was made in the SPS simulations to substitute the traditional “trial and error” method. This work is expected to be a valuable reference for designing electro-thermal coupling models of the SPS process.

## 2. Methods

### 2.1. Algorithm for Determination of PID Coefficients

The controllable and reproducible SPS process should have a stable temperature regulation, which requires the output temperature to be synchronized with the desired temperature profile. In SPS simulation, the PID controller is a useful tool to achieve this goal. It outputs the expected electric current density through the combination of proportional, integral and differential calculations of the input temperature error. The PID coefficients (or gains) are applied to amplify these input signals.

The classic linear PID combines three different controllers in parallel connection, which requires an error signal to output a control signal. Its form can be described as [17]:

(1) $$J(t)=K_{P}\Delta T(t)+K_{I}\int_{0}^{t}\Delta T(t)dt+K_{D}\frac{d\Delta T(t)}{dt}$$

where

(2) $$\Delta T(t)=T_{des}(t)-T_{out}(t)$$

in which *K_P_*, *K_I_* and *K_D_* are proportional, integral and derivative gains respectively; *t* is the sintering time; Δ*T*(*t*) is the temperature error obtained by the difference between the desired temperature *T_des_*(*t*) and output temperature *T_out_*(*t*); and *J*(*t*) is the output electric current density.

A given temperature profile in SPS simulation should have an expression of *T*(*t*). Normally, it is a linear function of time in most sintering cases, as a constant heating rate is often used. To establish the relation between the PID controller and the temperature profile in SPS, it is assumed that the PID coefficients depend on the instantaneous temperature (*T*) and heat rate (*dT*/*dt*) of a given temperature profile. This dependency can be assumed to be their linear combination:

(3) $$K_{i}(t)=\alpha_{i}T(t)\times \frac{dT(t)}{dt}+\beta_{i}\frac{dT(t)}{dt}+\gamma_{i}T(t)$$

in which subscript *i* can be *P*, *I* or *D*; *T*(*t*) and *dT*(*t*)/*dt* are, respectively, the instantaneous temperature and the heating rate of the desired temperature profile over time; and α, β and γ are variable parameters.

Generally, the temperature profiles vary in terms of their instantaneous temperature or heat rate features for different SPS processes. In order to find a universal temperature profile that can cover all the features of a given temperature profile, a “reference temperature profile” was constructed. Its expression is:

(4) Tref(t)=Aexp(−Bt)+C

(5) Tref(t)dt=ABt2exp(−Bt)

Here *A*, *B* and *C* are all temperature-related values. Straightforwardly, value A is the initial temperature and value C is the difference between the maximum and minimum temperature of the desired temperature profile. Value *B* is a time function dependent on the characteristics of the desired temperature profile. This reference temperature profile is an assumed one used to calibrate the PID coefficients of the desired temperature profile at different times. It has an ideal functional property which is that the heating rate decreases continuously from infinity at the initial temperature to zero at the maximum temperature. In other words, the heating rate can be any value at a given temperature in this reference profile. Thus, for an arbitrary point on the desired temperature profile we can always find an equivalence point that shares the same instantaneous temperature (*T*) and heating rate (*dT*/*dt*) with that point. This “equivalent point” has the same coordinate with this arbitrary point; that is, for an arbitrary point, the desired temperature profile is always tangent to its corresponding reference profile at this point. It can be noted that every point has its own distinctive reference profile. In this way, value B can be identified as a function of time by combining Equation (4) and the expression of the desired temperature profile.

Substituting Equation (4) into Equation (3) gives:

(6) Ki(t)=αiA2B×exp(−2Bt)t2+(αiABC+βiAB)×exp(−Bt)t2+γiA×exp(−Bt)+γiC

The reference temperature profile is commonly a continuous and smooth curve; the PID coefficients used for its temperature regulation should change gradually. A rapidly changing coefficient will result in an unstable output current density, which is not expected in the SPS process. Hence, it is reasonable to assume that the *dK_i_*/*dt* should be a small value close to zero:

(7) $$\frac{dK_{i}}{dt}\approx \frac{\Delta K_{i}}{\Delta t}\to 0$$

By deriving the first-order derivative of *K_i_* with respect to time, we get:

(8) dKidt=αiA2B×ddt[exp(−2Bt)t2]+(αiABC+βiAB)×ddt[exp(−Bt)t2]+γiA×ddtexp(−Bt)=0

That is,

(9) 2αiA2B×(B−t)×exp(−2Bt)t4+(αiABC+βiAB)×(B−2t)×exp(−Bt)t4+γiAB×exp(−Bt)t2=0

By removing the common factor, this becomes:

(10) 2αiA(B−t)×exp(−2Bt)+(αiC+βi)(B−2t)×exp(−Bt)+γit2×exp(−Bt)=0

Here, a variable *x* is introduced to simplify this equation.

(11) x=exp(−Bt)∈(0,1)

and, accordingly,

(12) $$t=-\frac{B}{\ln x}$$

Apparently, this *x* equals [*T_ref_*(*x*) *− C*]/*A*. It can be regarded as the heating progress, since value *A* is the initial temperature and value *C* is the difference between the maximum and minimum temperature of the temperature profile. Substituting Equations (11) and (12) into Equation (10) and removing the common factor, we get:

(13) $$2\alpha_{i}A\times \left[x+\frac{x}{\ln x}\right]+\left(\alpha_{i}C+\beta_{i}\right)\times \left[1+\frac{2}{\ln x}\right]+\gamma_{i}B\times \frac{1}{\ln^{2}x}=0$$

At this point, the numerical relation of parameters α, β and γ has been established for the construction of the PID coefficients of the reference temperature profile.

### 2.2. Construction of PID Controller for Desired Temperature Profiles

The parameters for constructing the optimal PID coefficients are not directly derived from the expression of the desired temperature profile; however, each point on it always has two important characteristics, temperature and heating rate, the linear combination of which is taken as the PID coefficients. They can be equivalent to the point on the “reference temperature profile”, which shares the same temperature and heating rate. As a result, these equivalent points should also correspond to the same PID coefficients, and they can be calculated by Equation (13). At this step, it is necessary to build a relation between the reference temperature profile and the desired temperature profile through the principle of equivalence. As mentioned before, the values *A*, *B* and *C* are the key to this connection. For a given temperature profile *T*(*t*), value *A* is determined as its initial temperature and value *C* is determined as the difference between its maximum and minimum temperature. The basic concept of equivalence is to find a point on the desired and reference temperature profiles where they share the same instantaneous temperature and heating rate. Therefore, value *B* can be determined by uniting the equality of *T*(*t*) and *T_ref_*(*t*) and the equality of *dT*/*dt* and *dT_ref_*/*dt*; then, the expression of value *B* as a function of time can be obtained.

Hence, all the unknown parameters have been solved in Equation (13). This equation can be mathematically regarded as a set of planes on the α-β-γ coordinate system. Each plane corresponds to a certain moment in the SPS process, and a suitable relation between α, β and γ values can always be found at any time of an SPS process. By substituting them back into Equation (3), the variable PID coefficients as functions of time are finally obtained. The initial PID coefficients were set as (400, 50, 5) in this work; the subsequent “trial and error” method can be replaced by this proposed method in the temperature regulation of SPS process. The selection of initial PID coefficients is flexible and not fixed, which brings more convenience to finding the optimal PID coefficients in the temperature regulation in SPS simulations. It should be noted that only the initial PID coefficients were selected in present work, since they were restricted to satisfy the zero approximation of their change rate of time. Then, by following the steps above, this developed numerical approach can be applied to search for the real PID coefficient values at each sintering time.

### 2.3. Criterion for Temperature Profile Fitting

In SPS simulation practice, the output temperature profile can hardly present an exact fit to the desired temperature profile. Some temperature deviation from the target profile is common and inevitable in the SPS process. One purpose of the stable temperature regulation is to drive the output temperature to keep the same pace with the prescribed temperature time-series data. The overshoot or undershoot is the frequently used indicator for evaluating the temperature stability at the onset of the insulation stage. Here we utilize the coefficient of determination (*R*^2^) to reflect the global fit degree in the temperature regulation process, which is:

(14) $$R^{2}=1-\frac{SSE}{SST}=1-\frac{\sum_{i=1}^{N}{\left(T_{i}^{out}-T_{i}^{des}\right)}^{2}}{\sum_{i=1}^{N}{\left(T_{i}^{out}-{\bar{T}}_{i}^{out}\right)}^{2}}$$

in which *SSE* is the sum of squared errors between the output temperature data and the desired temperature data and *SST* is the sum of squares of the differences between the output temperature data and their mean. The *R^2^* value characterizes the quality of a PID controller. The value range of *R*^2^ normally is typically [0, 1]. The closer it is to 1, the better the output temperature fits the formulated temperature data.

### 2.4. Configuration of Die–Sample Setup in SPS Simulation

**Figure 1:**
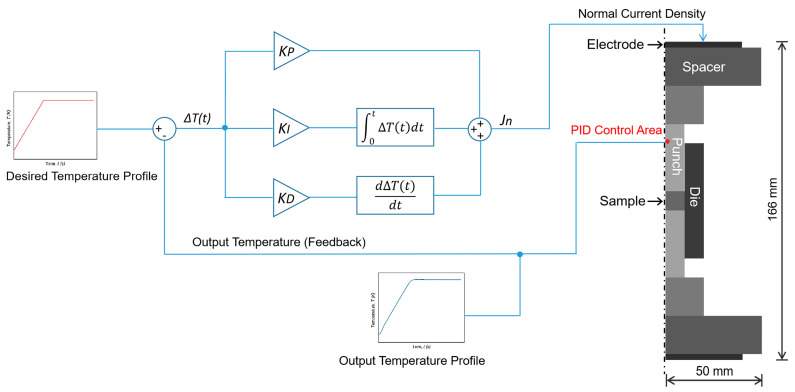
Schematic diagram of the SPS setup showing the component configuration and the control scheme.

Figure 1 shows the schematic diagram of the SPS setup, illustrated as an axisymmetric model, and the PID control scheme in the electro-thermal model. The linear PID control algorithm was embedded in this model through the mathematical interface in COMSOL (Version 5.6, 2020, COMSOL, Inc., Burlington, NJ, USA) to investigate the PID coefficients that are sensitive to the temperature changes. This setup mainly consisted of a sample (TiAl alloy), electrodes (stainless steel) and other graphite tools (spacers, punches and a die). The electro-thermal properties of the TiAl sample and graphite tools are given in Table 1. The electro-thermal simulation of the SPS process was based on the Joule heating model in order to predict the spatial temperature distribution in the SPS die–sample assembly, which follows the heat transfer relationship:

(15) $$\nabla \times (-\kappa \nabla T)+\rho C_{p}\frac{dT}{dt}=\vec{J}\vec{\times E}$$

in which *JE* is the heat generated by the electric current per unit volume, κ is the thermal conductivity, ρ is the density and *C_p_* is the specific heat. The heat was generated by the PID-controlled normal electric current density (*J_n_*) on the upper surface of the electrode, and the PID regulation area was selected in the middle (height) of the upper punch. A ground condition was set on the lower surface of the electrode, while all the lateral surfaces were considered to be electrically insulated.

It has been shown that the electric and thermal contact resistances (*ECR*/*TCR*) are of great importance to the electric and temperature field distribution [19,20]. In the SPS configuration reported here, *ECR* and *TCR* at all the internal interfaces were described as [20,21]:

(16) $$ECR=(-0.0057T+18.59)\times (-0.0079P+1.14)[m\Omega \cdot {\mathrm{cm}}^{2}]$$

(17) $$TCR=1.50\times 10^{-4}/(162-0.179T+1.07\times 10^{-4}T^{2}-2.43\times 10^{-8}T^{3})[K\cdot m^{2}/W]$$

The external pressure equaled zero in this case, as only the electro-thermal behavior of the sample was investigated. The cooling of the external electrodes could be simulated by convection flux with a convection coefficient of 200 W/(m^2^·K), and the graphite emissivity was determined to be 0.8 [22]. The natural convection flux of the graphite surface was neglected because this SPS process was conducted under vacuum conditions. Last, the samples used in this study were assumed to be dense to exclude the influences of porosity on the electro-thermal properties of materials.

## 3. Results and Discussion

### 3.1. Temperature Regulation in the SPS Devices

**Figure 2:**
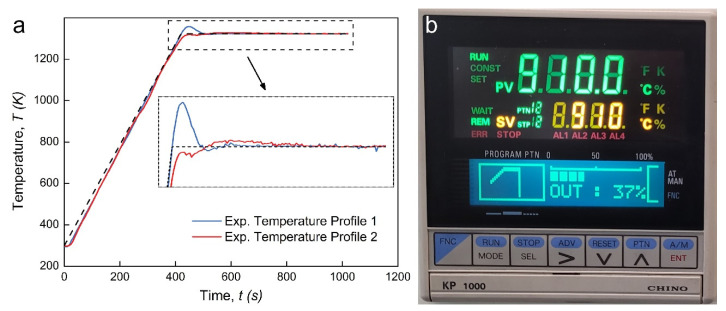
Proportional integral derivative (PID) temperature regulation in an SPS device: (**a**) the sintering temperature profiles and (**b**) the user interface of the PID temperature regulation.

With the assistance of an electric current field, the SPS device requires the accurate temperature output to achieve the anticipated grain growth and densification process in sintered materials. As a complex dynamic system, most SPS devices usually rely on their built-in PID controllers; however, the real temperature profiles are sometimes less than satisfactory. Figure 2 shows some examples of the sintering temperature curves of Fe–Ni-based alloys in our SPS experiments and the interface of temperature regulation in the SPS device.

In these experiments, the output temperature curves showed a non-negligible deviation from the preset values. Characterized by obvious overshoot (34.8 K) or undershoot (8.7 K), as well as by the oscillation at the holding stage, this SPS experiment may have failed to provide a stable and repetitive PID-assisted temperature regulation. This drawback may have been harmful to the microstructure and densification process of the material during the SPS experiment, which are very sensitive to the temperature cycle. The PID control algorithm, as the core of temperature regulation in the SPS device, and its improvement perhaps are of great importance to problem solving. Next, the feasibility of applying the PID control algorithm proposed above to SPS equipment is verified.

### 3.2. Regular Temperature Profile with Isothermal Insulation

To apply this proposed method, the common sintering cycle was firstly investigated in the SPS simulation. It contained a constant-rate heating stage followed by an isothermal holding stage. The PID-related temperature regulation of such a common sintering cycle, to some extent, can be estimated by prior experience. In this case, one can pre-set a set of the PID coefficients based on previous practices to receive some nearly ideal outcomes. Due to the simple profile features and the personalized sintering setup, only a few attempts are required in the “trial and error” step. In this stage, our numerical method was intended to stably regulate the simple temperature profile without the “trial and error” step.

Taking the temperature curve used in [16] as the target temperature profile, its expression of the heating stage can be considered approximately as:

(18) T(t)=3.33t+300,t∈(0,180)

Correspondingly, the assumed PID coefficients can be described as a function of time, which is:

(19) $$K_{i}(t)=(11.09t+1000)\alpha_{i}+3.33\beta_{i}+(3.33t+300)\gamma_{i}$$

Here the values A and C are fixed as 600 and 300, according to the previous description. The value *B* should be calculated by using the characteristic that the equivalent point has the same temperature and heating rate as a point on the desired profile, which is:

(20) $$B(t)=t\ln^{2}\left(\frac{t}{180}\right)$$

Correspondingly, in this case, the parameter relation used for constructing the PID coefficients at different times is:

(21) $$1200\alpha_{i}\times \left[x+\frac{x}{\ln x}\right]+\left(300\alpha_{i}+\beta_{i}\right)\times \left[1+\frac{2}{\ln x}\right]+\gamma_{i}\times 180x=0$$

**Figure 3:**
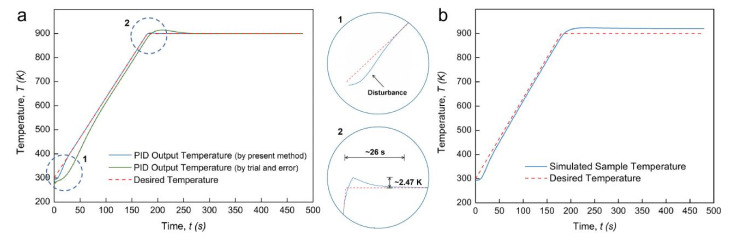
PID regulation outcome for regular temperature profile: (**a**) PID output temperature profile with enlarged views; (**b**) simulated sample temperature profile.

According to the target temperature profile, *x* equals *t*/180. The *K_P_*, *K_I_* and *K_D_* coefficients of this case can thus be determined as functions of time. Figure 3 shows the PID-controlled regulation results for this regular temperature profile and some detailed observations on their typical features. A worse temperature regulation result was observed when a traditional “trial and error” method was applied. Characterized by an outstanding global fit degree (*R*^2^) of 0.9991, the output temperature profile using the PID controller based on the present method maintained a high consistency with the expected profile. An abnormal disturbance at the very beginning of the heating ramp (shown with mark “1”) can be observed, with the initial temperature even lower than the starting temperature (300 K). This was perhaps caused by the singularity of the *K_i_* coefficient, which can be a large negative number when the time approaches zero. It should be noted that, at the beginning of the dwell time, a tiny temperature overshoot of about 2.47 K was generated and stabilized only after 26 s (shown with mark “2”). These local features, superior to other reports, clarify that the present numerical method for PID coefficients is of higher accuracy than the “trial and error” method. Figure 3b shows that the temperature evolution at the center of the TiAl sample had a small time lag between the punch and the sample at the heating stage but had an obvious excess at the dwell time. The TCR can be higher under low external pressure, leading to the heating delay of the sample at the heating stage and hindering the heat flux from passing out of the sample during the dwell time.

**Figure 4:**
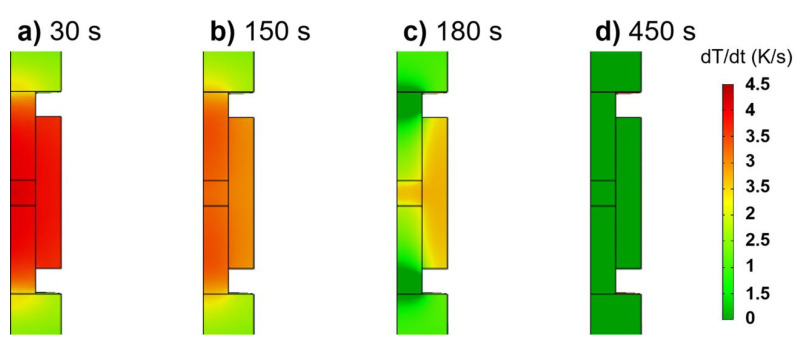
Typical heating rate fields from the punch-controlled PID regulation of regular temperature profile at different times: (**a**) 30 s; (**b**) 150 s; (**c**) 180 s; (**d**) 450 s.

The heating rate field under the regular profile regulated by the PID controller can be revealed as follows. At the beginning, punches, die and sample all have the maximal heating rate of about 4 K/s (Figure 4a); however, the punches have a slightly higher heating rate than the sample and the die during the late stage of the heating ramp (Figure 4b). The constraint effects of the contact resistance on the flow of the electric current and heat flux flow can be more remarkable as the temperature rises, leading to the heat accumulation at the punches. When the overshoot occurs at the beginning of the dwell time, the heat responsiveness shows distinct features between punches and sample (Figure 4c). The stabilization of the temperature first starts at the upper part of the punches, whereas the sample still experiences the maximum heating rate, but it decreases to about 2.5 K/s. After stabilizing PID, the temperature regulation finishes at the end of the dwell time, and the heating rate becomes zero in all the parts of the SPS setup (Figure 4d).

### 3.3. Continuous Temperature Profile with Cyclic Temperature Oscillation

One of the main purposes of SPS technology is to process various inorganic materials with the expected microstructure. It requires a thoughtful temperature cycle to realize the precise control of many processes, including grain growth and phase transition. Here, a more complex temperature profile with a cyclic temperature oscillation, rather than an isothermal holding, after the heating ramp stage was considered. This continuous temperature profile showed local maximum and minimum values and provided an example for the temperature regulation in advanced SPS processes.

**Figure 5:**
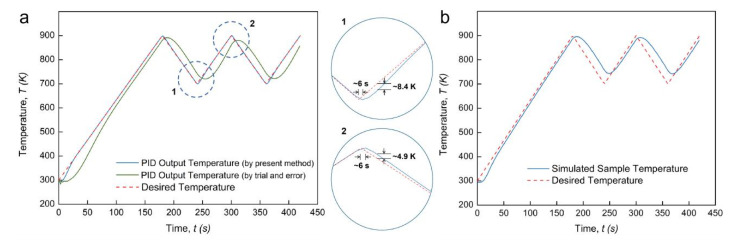
PID regulation outcome for continuous temperature profile: (**a**) PID output temperature profile with enlarged views; (**b**) simulated sample temperature profile.

Figure 5a shows the PID regulation results for the profile with a cyclic temperature oscillation. Obviously, the output temperature profile with the present method maintained a good match with the desired profile, with a high global fit degree (*R*^2^) of 0.9987 obtained; however, it is intuitive that the “trial and error” method is unable to provide a stable result. Two zoom-in pictures show the detailed features near the local max and min values. At these two extremums, the time delay in reaching the expected temperature value is about 6 seconds. For the local heating stage, a temperature undershoot of around 8.4 K appears; for the local cooling stage, a temperature overshoot of approximately 4.9 K appears. In Figure 5b, the simulated sample temperature shows an obvious deviation from the expectation profile in the temperature cyclic oscillation stage. The sample temperature can hardly reduce to the local minimum value (700 K). This hysteresis of the temperature change during the local cooling is apparently more severe than during the local heating.

**Figure 6:**
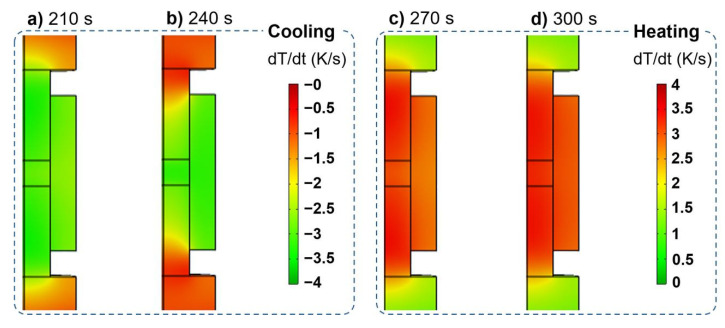
Typical heating rate fields from the punch-controlled PID regulation of continuous temperature profile at different times: (**a**) 210 s; (**b**) 240 s; (**c**) 270 s; (**d**) 300 s.

Figure 6 shows the heating responsiveness maps indicating the temperature hysteresis of the sample during the temperature cyclic oscillation stage. During the local cooling stage, the punches have a maximum cooling rate of about 3.5 K/s, slightly higher than that of the sample (Figure 6a). The electric current at this stage is sharply decreased. Thus, the SPS setup is subjected to the cooling process. Due to the surrounding of the high TCR interfaces, it is difficult for the heat flux to flow out of the TiAl sample, and an obvious temperature hysteresis occurs. The cooling in SPS can generally be realized by gas cooling in chamber. The gas flow can be used to rapidly cool down the SPS setup and a PID controller is perhaps able to control this cooling process by regulating the gas flow rate. When the temperature reaches the local minimum value, the punches finish their cooling process at first, and a significant temperature difference is generated between the TiAl sample and the punches (Figure 6b). This causes the sample to maintain a high cooling rate and have an obvious temperature hysteresis. Like the initial heating ramp, the heating rate is maximal in the punches at about 3.5 K/s during the local heating stage (Figure 6c). Moreover, the punches and the sample maintain a close heating rate until the temperature reaches the maximum local value (Figure 6d). As a result, there is a synchronized heating process between the punches and the sample.

### 3.4. Non-Continuous Temperature Profile with Sudden Temperature Change

To further investigate the general feasibility of applying this numerical method to constructing a PID controller, a non-continuous temperature profile was considered. In this case, the temperature is subjected to a sudden change after each isothermal holding for a period of time. The temperature change can be considered as ultra-fast during this process. Conventional SPS equipment may withstand the ultra-high electric current required to realize such a sudden temperature rise; however, achieving a stable regulation of this kind of temperature profile is of great importance. This temperature profile has many application scenarios in SPS and a feasible way can be offered to study the sintering processes of materials under such situations. For instance, thermally cycled sintering can help accelerate the densification during the SPS process [23]. In this case, a rapid temperature change for promoting the cyclic phase transition is necessary for the acceleration of densification process [23]. The strong and tough ceramics with ultra-fine grained structure can be prepared by a multi-stage spark plasma sintering process, which requires a quick temperature increase after each sintering stage [24,25].

**Figure 7:**
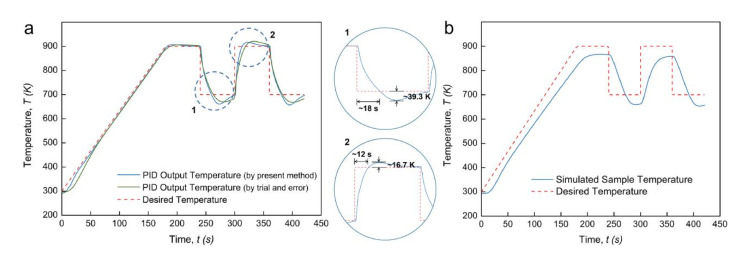
PID regulation outcome for non-continuous temperature profile: (**a**) PID output temperature profile with enlarged views; (**b**) simulated sample temperature profile.

Figure 7 shows the PID-controlled regulation results for this non-continuous temperature profile. Intuitively achieving a suitable temperature regulation by using the classic PID controller is not expected, whether it is through “trial and error” or the present methods. As shown in Figure 7a, a long time delay of about 18 s and an unexpected temperature undershoot of about 39.3 K take place during the rapid cooling process. In addition, a long time delay of about 12 s and a high-temperature overshoot of about 16.7 K take place during the rapid heating process. All these deviations cannot be stabilized within a short time range. Figure 7b further shows the simulated sample temperature profile under this PID regulation condition. During the heating ramp and the thermal cycle stage, the sample temperature is far from the desired profile. It can be confirmed that the PID controller constructed by the proposed numerical method is not suitable for the regulation of non-continuous temperature profiles with sudden temperature changes. One of the possible reasons is that the PID coefficients were assumed to be continuous time functions. The main objective, in these cases, is to realize a short time delay and small overshoot or undershoot when a rapid temperature change happens. Utilization of the fraction order PID controller (FOPID or PI^λ^D^μ^) could be a promising solution to this problem [26,27], as two additional adjustable parameters result in superior control performance. Beyond the scope of the present work, our future work will focus on obtaining the stable regulation of the non-continuous temperature profiles with thermally cyclic stages by using a FOPID controller.

### 3.5. Limitations of the Developed Numerical Method for Construction of PID Coefficients

The proposed numerical method for construction of the PID coefficients has provided some satisfactory outcomes for temperature regulation during the SPS process. This work is an initial step towards the generalized design of PID controllers for SPS simulations. However, this method was developed without detailed consideration of the physical role of each component of the PID controller in the dynamic system. Generally, the proportional part is associated with the present error in the system, the integral part is related to the error history in the system, and the differential part is related to the trend of the error change in the system [28]. In the developed numerical method, the influence of each component on the temperature regulation in the SPS simulation was not distinctly emphasized. Although the utilized approximations are reasonable based on the features of the desired temperature profiles and the linear features of the classical PID controller, the general applicability of the obtained numerical method to arbitrary temperature profiles in the SPS processes still needs to be further verified.

## 4. Conclusions

Tuning the PID coefficients with the common “trial and error” method often involves some time-consuming procedures. A numerical method based on the principles of variable coefficients and temperature equivalence was proposed in the present study, aiming to construct a PID controller for SPS simulations. When the designed PID controller was applied to regular temperature profiles, the overshoot at the beginning of dwell time was significantly reduced. When the continuous temperature profile with a cyclic oscillation stage was applied, the PID-regulated temperature profile still followed the prescribed profile very accurately. However, the classical PID controller designed here did not offer satisfactory regulation for the non-continuous temperature profile, which could possibly be improved by adopting a fractional order PID controller. Although there are some limitations, the proposed numerical method provides an attractive approach for a generalized rule in constructing efficient PID controllers for SPS simulations. This work demonstrates that finding the optimal PID coefficients by building their numerical relationships is an effective way to improve temperature regulation in electro-thermal simulations of SPS processes.

## Figures and Tables

**Table 1:** The electro-thermal properties of materials used in this spark plasma sintering (SPS) setup [16,18].

Material	Heat Capacity, *C_p_* (J·Kg^−1^·K^−1^)	Thermal Conductivity, κ (W·m^−1^·K^−1^)	Electrical Resistivity, ρ_e_ (Ω·m)	Density, ρ (kg·m^−3^)
Graphite	34.27 + 2.72*T* − 9.6 × 10^−4^*T*^2^	123 − 6.99 × 10^−2^*T* + 1.55 × 10^−5^*T*^2^	1.70 × 10^−5^ − 1.87 × 10^−8^*T* + 1.26 × 10^−11^*T*^2^ − 2.44 × 10^−15^*T*^3^	1904 − 0.0141*T*
Electrode	446.5 + 0.162*T*	9.99 + 0.0175*T*	(50.2 + 0.0838T − 1.76 × 10^−5^*T*^2^) × 10^−8^	7900
TiAl	583 + 9.40 × 10^−2^*T*	9.47 + 1.19 × 10^−2^*T*	4.06 × 10^−7^ + 6.45 × 10^−10^*T*	3900

## Data Availability

Data sharing is not applicable.
